# Bacterial Biofilm Inhibition: A Focused Review on Recent Therapeutic Strategies for Combating the Biofilm Mediated Infections

**DOI:** 10.3389/fmicb.2021.676458

**Published:** 2021-05-12

**Authors:** Ramanathan Srinivasan, Sivasubramanian Santhakumari, Pandurangan Poonguzhali, Mani Geetha, Madhu Dyavaiah, Lin Xiangmin

**Affiliations:** ^1^Fujian Provincial Key Laboratory of Agroecological Processing and Safety Monitoring, School of Life Sciences, Fujian Agriculture and Forestry University, Fujian, China; ^2^Key Laboratory of Crop Ecology and Molecular Physiology (Fujian Agriculture and Forestry University), Fujian Province University, Fujian, China; ^3^Department of Biochemistry and Molecular Biology, School of Life Sciences, Pondicherry University, Puducherry, India; ^4^Department of Microbiology, M.R. Government Arts College, Tamil Nadu, India; ^5^PG Research and Department of Microbiology, St. Joseph’s College of Arts and Science (Autonomous), Tamil Nadu, India; ^6^Key Laboratory of Marine Biotechnology of Fujian Province, Institute of Oceanology, Fujian Agriculture and Forestry University, Fujian, China

**Keywords:** antibiotics, bacterial biofilm, biofilm inhibitors, biofilm mediated infections, multidrug resistance, persistence, therapeutic strategies

## Abstract

Biofilm formation is a major concern in various sectors and cause severe problems to public health, medicine, and industry. Bacterial biofilm formation is a major persistent threat, as it increases morbidity and mortality, thereby imposing heavy economic pressure on the healthcare sector. Bacterial biofilms also strengthen biofouling, affecting shipping functions, and the offshore industries in their natural environment. Besides, they accomplish harsh roles in the corrosion of pipelines in industries. At biofilm state, bacterial pathogens are significantly resistant to external attack like antibiotics, chemicals, disinfectants, etc. Within a cell, they are insensitive to drugs and host immune responses. The development of intact biofilms is very critical for the spreading and persistence of bacterial infections in the host. Further, bacteria form biofilms on every probable substratum, and their infections have been found in plants, livestock, and humans. The advent of novel strategies for treating and preventing biofilm formation has gained a great deal of attention. To prevent the development of resistant mutants, a feasible technique that may target adhesive properties without affecting the bacterial vitality is needed. This stimulated research is a rapidly growing field for applicable control measures to prevent biofilm formation. Therefore, this review discusses the current understanding of antibiotic resistance mechanisms in bacterial biofilm and intensely emphasized the novel therapeutic strategies for combating biofilm mediated infections. The forthcoming experimental studies will focus on these recent therapeutic strategies that may lead to the development of effective biofilm inhibitors than conventional treatments.

## Introduction

Biofilms are severe health concerns due to their multidrug resistance abilities, host defense, and other stresses (De La Fuente-Nunez et al., 2013). Therefore, it leads to chronic bacterial infections worldwide (Subhadra et al., 2018; Sharma et al., 2019). Bacterial biofilm is a multifaceted structure of communities with diverse bacterial colonies of cells in a group (Kostakioti et al., 2013). Biofilm referred to the intricate three-dimensional (3-D) aggregation of bacteria attached to a surface and buried inflexibly in an Extracellular Polymeric Substance (EPS) matrix. Bacteria form biofilms in every substratum, and their associated infections in plants, animals, and humans (Lebeaux et al., 2014; Padmavathi et al., 2017; Kannappan et al., 2020). Besides, biofilms also play destructive roles in industrial pipelines corrosion (Lenhart et al., 2014). Bacterial biofilms can attach to various materials such as metals, glass surfaces, plastic wares, tissues, and clinical devices. Bacterial communities also produce biofilm, especially on all medical implants, including vascular grafts, heart valves, intrauterine devices, pacemakers, prosthetic joints, catheters, sutures, and contact lenses to acute infections (Kannappan et al., 2017b).

Bacteria inside the biofilm can also withstand harsh conditions and hold secreted polymers such as polysaccharides, extracellular DNA (e-DNA), proteins, and amyloidogenic proteins (Sharma et al., 2019). The pathogenesis and persistence of bacterial pathogens are dependent on the formation and maintenance of intact biofilms (Xu et al., 2000; Stewart and Costerton, 2001; Pang et al., 2013; Wilkins et al., 2014). Bacterial cells swathed in the biofilm are up to 1000 fold resistant to antibiotic agents. In this biofilm form, bacteria are more resistant to various antibacterial and chemical treatments. Biofilms offer the guard to the bacteria from pH, nutrients deficiency, and mechanical forces (Bryers, 1993; Sutherland, 2001; Singh et al., 2017). Therefore, the biofilm matrix gives additional resistance to bacteria, leading to bad bug’s infections like drug resistant bacteria.

Novel biofilm inhibitors have been investigated from a various sources in order to prevent biofilm formation and eliminate persistent biofilms. Although the research focused on identifying compounds able to target and inhibit this biofilm mode of bacterial growth is explicitly still inadequate (Peng et al., 2015; van Tilburg Bernardes et al., 2015; Kannappan et al., 2017a; Srinivasan et al., 2018). As a result, new therapeutic options are needed for controlling the biofilm associated infections. At present, the study of biofilm and its strategies to eliminate without any resistant development is one of the utmost significant fields of research. Several reviews on biofilm inhibitors have already been reported, but our review mainly focuses on significant novel strategies to control biofilm mediated bacterial infections.

## Biofilm Formation

**FIGURE 1:**
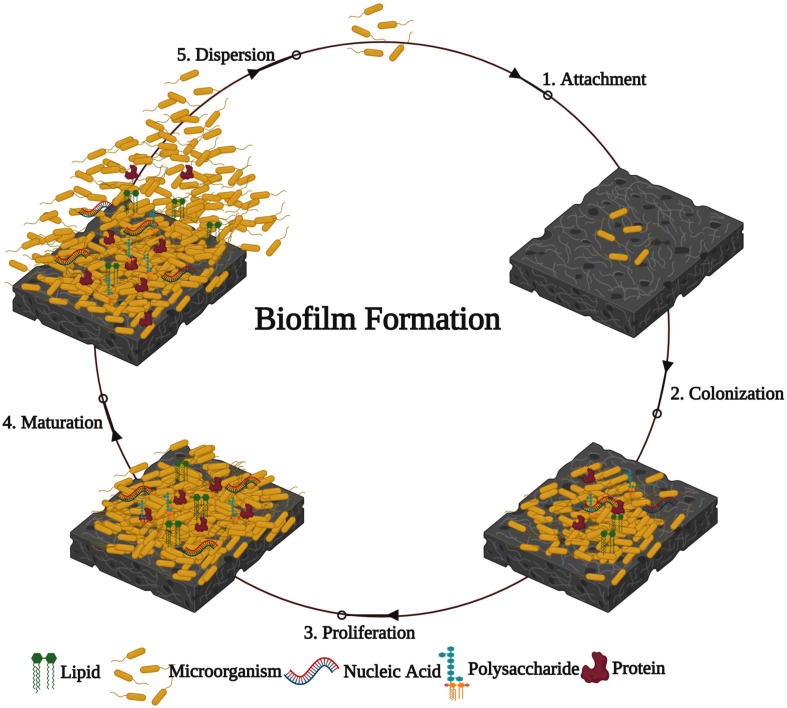
Developmental stages involved in bacterial biofilm formation.

Generally, biofilm formation by bacterial pathogens on any substratum/layer involves five major stages (Kostakioti et al., 2013; Yin et al., 2019). (1) Attachment: at an initial stage, free-swimming planktonic cells reversibly attach to the biotic or abiotic surfaces through weak interactions such as acid-base, hydrophobic, Van der Waals, and electrostatic forces. (2) Colonization: bacterial pathogens irreversibly attach to the surface through stronger interactions such as collagen-binding adhesive proteins, lipopolysaccharides, flagella, and pili. (3) Proliferation: the multilayered bacterial cells are profoundly accumulated, and the enormous amounts of EPS are produced. (4) Maturation: the attached multilayered bacterial cells grown into the matured biofilm with the typical 3D biofilm structure. (5) Dispersion: after the complete development of biofilm, it is disassembled or dispersed using mechanical and active processes (Figure 1).

### Characteristics of Biofilm Formation

The formation of biofilm is a progressive process. Primarily, bacterial cells move onto a surface and adhere reversibly to the surface. In the second step, irreversible adherence occurs with the microcolonies expansion that produces an EPS matrix. Subsequently, the progress of the mature 3-D biofilm architecture emerges. Matured biofilms are more resistant to the host immune defenses and the action of antibacterial agents. During the dispersal of biofilm, the cells endure lysis and discharge from the biofilm community. Inside the host, bacteria produce biofilm on a biotic or an abiotic layer. The abiotic surface is typically coated with proteins or other biological molecules, forming a habituation film that changes cells adhesion. In biofilm formation, host cells can develop a fundamental part, and their components can be assimilated into the biofilm matrix (Lynch and Robertson, 2008; Romling and Balsalobre, 2012).

### Components in the Biofilm Matrix

Biofilms are a group of microorganisms in which microbes produced EPS such as proteins (<1–2%), polysaccharides (1–2%), DNA (<1%), and RNA (<1%). In addition to these components, water (up to 97%) is the key portion of biofilm, distributed in a non-homogenous pattern and mainly accountable for the movement of nutrients inside the biofilm matrix (Batoni et al., 2016; Nazir et al., 2019). The capability to build and conserve an organized biofilm community mainly depends on EPS matrix components (Sutherland, 2001; Branda et al., 2005; Limoli et al., 2015). The EPS in the biofilm matrix commands a charter for the biofilms. The biofilm inhabitants are always shielded from the atmosphere (competitive microbes, temperature, host cells, antimicrobials, and desiccation) while also having access to nutrients and the capacity to react environmental changes. Bacteria generate multiple types of EPS to handle these needs in different ways. EPS can help the bacteria to adhere on many different surfaces and hosts; provide protection from the environment and reservoirs for nutrient acquisition (Ramanathan et al., 2018; Kannappan et al., 2019a).

### Role of EPS in Biofilm Formation

EPS is a superglue that accounts for the biofilm communities slimy nature and is a complex blend of biopolymers, including polysaccharides, proteins, e-DNA, and phospholipids (Nazir et al., 2019). In general, EPS composition changes with the type of pathogens, biofilm age, and environmental conditions (desiccation, pH, oxygen, nitrogen, temperature, and nutrients availability) (Mayer et al., 1999; Kostakioti et al., 2013). The bacteria existing in biofilm suggest that they can respond to their surroundings by modifying their EPS composition and adhesion. EPS provides a physical framework for the attachment among cells and surfaces. It also acts as a blockade between biofilm cells and surroundings (Mitchell et al., 2016). It protects microbes from antimicrobial compounds, chemicals, desiccation, radiation, and unfavorable environmental conditions. They are also cherishing bacterial cells inside the biofilm with a constant supply of nutrients and keeping their capability to respond environmental variations. As related to a protein in EPS composition, polysaccharides are extremely sticky and fundamental for biofilm maintenance and its environment. Similarly, proteins from EPS matrix modify the cell wall assets, adherence, virulence, and morphogenesis; protect cells from harmful conditions and phagocytes (Chaffin et al., 1998; Bridier et al., 2011). Another vital component of EPS is e-DNA, which increases biofilm structural integrity, exchange of genetic information, nutrients provision, biofilm stability, and drug resistance (Donlan, 2002; Martins et al., 2010).

### Biofilm Mediated Bacterial Infections

**FIGURE 2:**
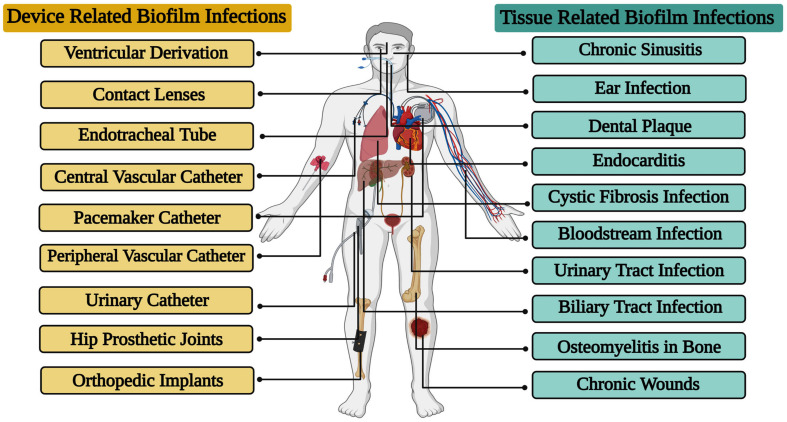
Various types of tissue and device related biofilm infections caused by bacterial pathogens.

The level of clinical care has advanced dramatically over the few years, but bacterial biofilm infections continue to pose a significant threat to public health. Hoiby, Lam and his colleagues were the first to identify a direct correlation between the formation of biofilms and recurrent infections, especially with *Pseudomonas aeruginosa* in cystic fibrosis patients (Hoiby, 1977; Lam et al., 1980). The decades that followed embraced the idea that biofilms are an important source of tissue related infections (Lebeaux et al., 2013). There are various sites in the human body where biofilm infections may occur due to either a pre-existing condition or a hospital acquired infection. Further, tissue related bacterial biofilm infections have been noted to occur more often in immunocompromised patients, and patients with underlying chronic illness such as cardiovascular disease, diabetes, skin barrier breakage, cancer, or especially if the infection is severe or starts early in the course of the illness (Sivaranjani et al., 2018). In addition, it was understood that the usage of different forms of embedded medical devices would favor adhesion and the colonization of bacteria, resulting in infections (Marrie et al., 1982; Donlan and Costerton, 2002). Further, several types of embedded medical devices are associated with the development of bacterial biofilms (Figure 2). More, ventilator pneumonia, central line bloodstream infections, urinary, pacemaker, and peripheral vascular catheter infections are the utmost common device related bacterial biofilm infections (Kamaruzzaman et al., 2018; Kannappan et al., 2019b).

### Biofilm in Antibiotic Tolerance and Persister Formation

Usually, chronic tissue and device related bacterial infections are difficult to treat because the patient is exposed to the risk of recurrence (Lewis, 2007). Bacterial biofilms can spread to other parts of the body or around the infection source if planktonic bacteria originate from the biofilm. Planktonic bacteria may be eradicated by combined action of host immune responses and antibiotics. However, a subset of biofilm bacteria those are not destroyed by the antibiotic treatment and can able to trigger the recurrence of infection (Lebeaux et al., 2014).

**FIGURE 3:**
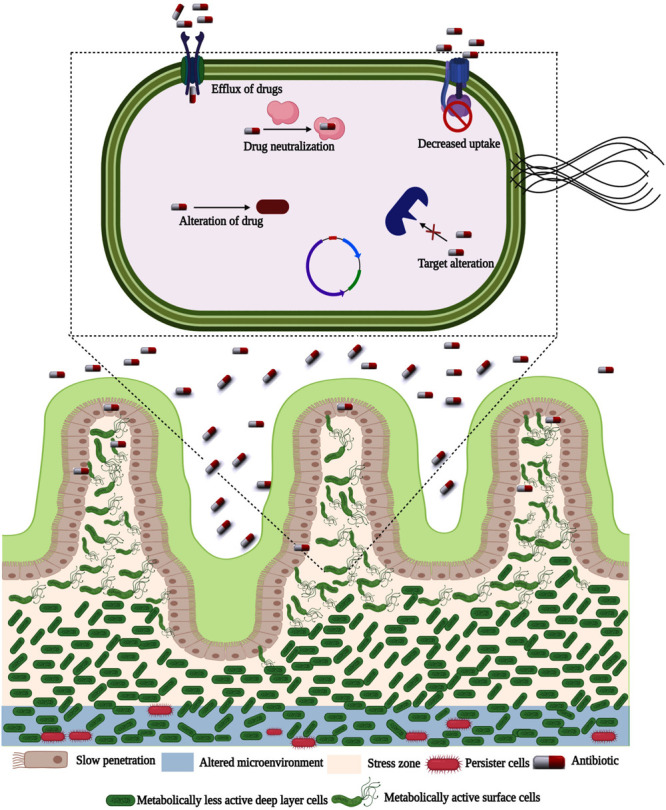
Antibiotic resistance and persister formation in bacterial biofilm. In the presence of EPS, antibiotic penetration is slowed. In reaction to antibiotic stress, certain bacteria in the biofilm alter their behavior. To resist biofilm eradication, the microenvironment in deeper parts is changed. Biofilms have a more concentration of persister cells in the altered microenvironment.

Inside the biofilm, bacterial cells reveal morphological, and physiological changes assisted by differential gene expression due to the gradient in toxic components, diffusible gasses, or nutritional pressure (Stewart and Costerton, 2001). Depleted oxygen and nutrients within biofilm stimulate asynchronous growth, which exhibits variations in the level of gene expression and may lead to drug tolerance. Phenotypic variety of bacteria within biofilm augments greater coordination, empowers genes for reprogramming, and involves the efflux of toxins, lipid biosynthesis, iron sequestration, DNA repair, and host immune modulation, etc. (Li et al., 2016). It provides persistence and selective dissemination of resilient cells enduring stress. Nowadays, resistance to antibacterial agents is the most crucial cause of non-effective therapy of biofilm-associated bacterial infections. The reason behind increased antibiotic resistance of bacteria is (1) Difficulty for the diffusion of antibiotics into the biofilm and electrostatic charge of the EPS, which attract oppositely charged antibiotics; (2) A slower growth rate; (3) Variations in phenotype acquired by bacteria forming biofilms and (4) Inactivation of antibiotics by enzymes secreted by bacteria (Figure 3; Lewis, 2001; Lewis, 2008, 2010; Sharma et al., 2019).

Several promising mechanisms are underlying the phenotypic resistance, which may be influenced by the type of antibiotic treatment and the host, growth rate of biofilm, transformed metabolism, and the presence of an oxygen gradient that prevents the action of some antibiotics (Li et al., 2016; Cheng et al., 2019). Besides, biofilms contain a great population of persister cells, which endure against antibiotics treatment. A limited dispersal of antibiotics into biofilms has been proposed, but in most occurrences, no direct evidence has been provided (O’Toole et al., 2000; Lewis, 2010).

### Signaling in Biofilm Formation: How Bacteria “Talk to Each Other”

Bacterial cells in biofilm communicate with each other and coordinate their behavior through the signal molecules. This cell-cell communication system is called Quorum Sensing (QS), which goes beyond bacterial cell density (Miller and Bassler, 2001). Mostly, roles of QS are classified into four kinds such as (1) Cell maintenance and division (exoenzymes and siderophores production), (2) Horizontal gene transfer (conjugation), (3) Host-pathogen interactions (antibiotic and bioluminescence production), and most importantly (4) Behavior (movement and biofilm formation). The QS mediated biofilm formation has well documented in several Gram-negative and Gram-positive bacterial pathogens (Carniol and Gilmore, 2004; Labbate et al., 2004; Kong et al., 2006; Longo et al., 2014; Brindhadevi et al., 2020).

Generally, QS facilitates the physiological status of the microbial population and controls the biofilm formation through signal molecules called *N*-acyl-homoserine lactones (AHL) or auto-inducing peptides (AIP) in Gram-negative and Gram-positive bacterial pathogens, respectively (Whitehead et al., 2001; Papenfort and Bassler, 2016; Rama Devi et al., 2016; Bhatt, 2018). According to the bacterial species, varieties of AHL exist in Gram-negative bacterial pathogens, and some may vary as the strain varies. Usually, the AHL are synthesized by AHL synthase gene *luxI*. The *luxI* gene is transcriptionally expressed to the basal level at low population density. Hence, the AHL molecules are scattered in the field. At the high cell density, the LuxR family of receptor proteins senses the AHL molecules. Then, the signal molecule attaches to the receptor protein until AHL hits a particular threshold concentration. At that time, the activated LuxR-AHL complex forms multimers with other activated LuxR-AHL complexes. Finally, these multimers control the transcription of QS regulated biofilm formation in several bacterial pathogens (Rasmussen and Givskov, 2006).

## Recent Therapeutic Strategies for Biofilm Inhibition

Besides the conventional antibiotics, certain promising underlying strategies extended by the prevailing biofilm inhibitors hinder the biofilm formation and reduce microorganisms’ virulence. Most biofilm forming microorganisms are responsible for 80% of human infections (Shunmugaperumal, 2010) and their ill-health. Due to the EPS matrix of the biofilm, they resist the immune system of humans. Some antimicrobial peptides like defensins or existence biofilm inhibitors have an extensive part in acting upon the matrix (Lewis, 2001). However, many novel and interesting tactics or lines of attack combating against biofilms were identified, and their progression at the current scenario described in detail.

### Quorum Sensing (QS) Blockage Strategy

**FIGURE 4:**
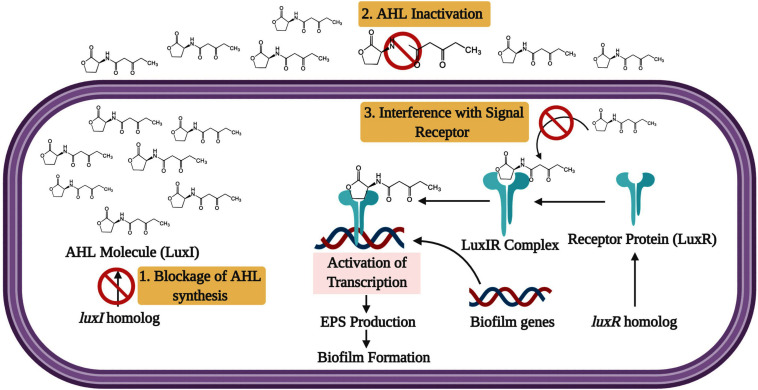
The inhibition approaches for LuxI/LuxR QS system. The signaling molecule AHL is produced by the *luxI* synthase gene and freely diffuses from each cell. When critical concentration is reached, the synthesized signal molecules diffuse back inside the bacterial cell and binds with LuxR. Then the QS transcription is activated by the LuxR-AHL complex. The target stages for inhibition are (1) Blockage of AHL molecule synthesis, (2) Degradation of the AHL molecule, and 3. Interference with the signal receptor.

Considering the QS system as the noteworthy comportment of biofilm synthesis by microorganisms, many researches contributed to recognizing the QS system’s blockage as a vital strategy to prevent biofilm. More, bacterial pathogens in the host can activate the QS signals for biofilm formation and virulence factors production. Therefore, inhibiting this bacterial communication through QS inhibitors makes the bacterial pathogens more susceptible to the host immune system and antibiotic responses (Ravindran et al., 2018; Jiang et al., 2019; Li et al., 2020). Consequently, it facilitates the targeting of QS as a therapeutic target for controlling biofilm mediated bacterial infections. The phenomenon of down-regulating or silencing the QS system is referred to as quorum quenching. Generally, blocking the QS system of Gram-negative bacterial pathogens can be done through three essential strategies: 1. Blocking the AHL molecule biosynthesis, 2. AHL inactivation or degradation, and 3. Interference with the signal receptor (Figure 4).

### Hindering the AHL Signal Molecule Biosynthesis

Previously, the *in vitro* analysis has been performed on the catalysis of AHL molecule biosynthesis in sequentially ordered reaction. The S-adenosyl methionine (SAM) is used as the amino donor to produce homoserine lactone ring moiety. An adequately charged acyl carrier protein (ACP) is used as the precursor for producing the acyl side chain of the AHL signal (Parsek et al., 1999; Hentzer and Givskov, 2003; Li et al., 2016). Other studies made by Zano et al., 2013 and Masevicius et al., 2016 have shown that several Gram-negative bacterial pathogens can synthesize the S-adenosyl-L-methionine (AdoMet) as the primary methyl donor for several methylation processes. Zano et al. (2013) have revealed that this AdoMet may also act as a precursor for the production of two different QS signal molecules; therefore, targeting the hindrance of AdoMet can lead to inhibiting the biofilm formation in various Gram-negative bacterial pathogens. So, QS inhibitors that target AHL molecule biosynthesis can be developed using knowledge of signal generation. Various analogs of SAM have been continuously revealed to be an effective inhibitor of AHL molecule biosynthesis. Some antagonists of SAM, such as S-adenosylcysteine, and S-adenosylhomocysteine have shown to ensure the capacity to effectively inhibit the AHL synthesis, which is facilitated by the *P. aeruginosa* RhlI protein (Parsek et al., 1999). Further, Christensen et al. (2013) have screened the QS inhibitors to target the AHL molecule biosynthesis against Proteobacteria such as *Burkholderia mallei* and *Yersinia pestis* through high-throughput screening. Some earlier experiments have shown that macrolide antibiotics such as azithromycin and erythromycin administered at sub inhibitory concentrations have the capacity to suppress the *P. aeruginosa* AHL molecule biosynthesis and thereby inhibited their virulence factors and biofilm formation (Sofer et al., 1999; Pechere, 2001; Tateda et al., 2001).

### AHL Signal Molecule Biodegradation or Alteration

**FIGURE 5:**
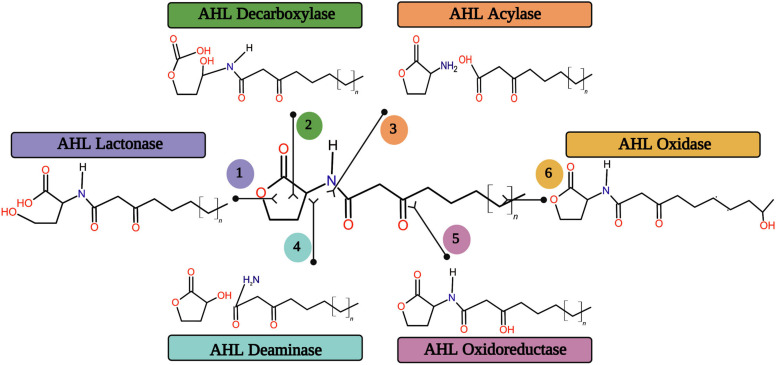
The six major quorum quenching enzymes on the degradation or alteration of AHL signal molecule.

Searching for enzymes capable of breaking down the AHL signal molecules is a promising strategy to eradicate the biofilm mediated bacterial infections altogether. AHL molecules are enzymatically destroyed by various forms of enzymes, eliminating AHL accumulation in the system. Generally, the enzymatic degradation or alteration of AHL signal molecules can be catalyzed by six major classes of enzymes rendering to their catalytic sites (Figure 5).

The AHL lactonases are capable of opening the homoserine lactone ring by way of breaking the bond on the leftward of the double bonded oxygen. Further, the enzyme decarboxylases are also capable of doing the same by way of breaking the bond on the rightward of the double bonded oxygen without disturbing the rest of the AHL molecule structure. AiiA 24B1, the product of the *aiiA* gene from *Bacillus* spp. 24B1, hydrolyzes the lactone ring in the homoserine moiety of AHL and which is one of the first identified and well characterized AHL lactonase (Dong et al., 2000). A recent study made by Shastry et al. (2019) has revealed the biofilm inhibitory efficacy of AHL lactonase enzyme on the *Aeromonas hydrophila* biofilm formation. The AHL acylase is a family of enzyme that corresponds to the Ntn hydrolase superfamily. It hydrolyzes the AHL signal molecules, as their names imply (Utari et al., 2017). The AHL acylase enzyme was first exposed in the *Variovorax paradoxus* strain VAI-C. It hydrolyzes the amide bond between the homoserine lactone and acyl side chain in AHL molecules, releasing homoserine lactone (HSL) and free fatty acid (Leadbetter and Greenberg, 2000). Several AHL acylase enzymes were identified from different bacterial sources for their biofilm inhibitory potential (Paul et al., 2009; Christiaen et al., 2014). The acyl side chain may also be cleaved from the HSL ring by deaminase but at a different location. Deamination encodes final products as an acyl side with NH_2_ and homoserine lactone with OH (Kose-Mutlu et al., 2019). The AHL oxidase catalyzes the carbon atoms oxidation in acyl chains of AHL signal molecules (Gao et al., 2013). The AHL oxidoreductases oxidize or reduce the carboxyl group of the third carbon to attack the side chain of AHL molecules. This type of enzymes does not break down the signal, but rather it alters the AHL molecule, thereby modifying the binding efficacy of receptor proteins with signal molecules (Vogel and Quax, 2019). The AHL oxidoreductase enzyme has recently been reported for its inhibitory potential on the autoinducer-2 mediated biofilm formation in Gram-negative bacterial pathogens by modifying the AHL signal molecule (Weiland-Brauer et al., 2016).

### Interference With Receptor Proteins by Analog Compounds

The membrane receptors can be interrupted by binding the antagonistic molecules so that the receptors are unavailable to bind with AHL signaling molecules. If there is no signal recognition, then there is a variation in the bacterial population’s physiological behavior, especially in reducing biofilm activity, less virulence, and low antibacterial tolerance. Furanone has been first identified analog compound as the potent QS inhibitor, which effectively inhibits the biofilm formation of *Staphylococcus epidermidis* (Hume et al., 2004). Further, the study of Lonn-Stensrud et al. (2009) has highlighted the antagonistic activity of furanone with a drastic interlude in the QS signaling, isolated from the marine algae, *Delisea pulchra*. The QS system of *Vibrio harveyi* was silenced by a marine strain *Halobacillus salinus*, which synthesize an antagonistic molecule that suppress the activation of *lux* gene and therefore hindering the signaling molecule biosynthesis (Teasdale et al., 2009). Similarly, in yet another study by Choi et al. (2012), honaucins was synthesized by *Leptolyngbya crosbyana*, which affects cell-to-cell communication by inhibiting the QS. Furthermore, bacterial communication of QS can also be blocked by the fungal metabolite such as patulin and penicillic acid from different *Penicillium* strains, respectively. For instance, the reports of Wagner et al. (2004); Rasmussen et al. (2005); Abraham (2005) have revealed that the QS in *P. aeruginosa* could be inhibited by the penicillinic acid and patulin. The antagonistic potential of phytol on biofilm mediated infections in *Serratia marcescens* was confirmed through *in vitro* studies by Alexpandi et al. (2019) and Srinivasan et al. (2016). Further, the pre-clinical trial has been performed in a mouse model and confirmed these antagonistic molecules activity as efficient QS inhibitors. Similarly, studies made by Srinivasan et al. (2017); Arunachalam et al. (2018) have revealed the biofilm inhibitory potential of antagonistic molecules such as phytol and geraniol on *S. marcescens* associated acute pyelonephritis infection and *S. epidermidis* associated endocarditis infection in animal models, respectively.

Apart from these three main strategies, some antimicrobial peptides could inhibit the QS system either by affecting the signal molecules transport within or outside the cell, thereby affecting the signal transduction cascade and biofilm formation. More, a newer tool in recent research is employed to block the expression of the *luxS* gene of *Escherichia coli* during QS signaling to reduce the biofilm formation by CRISPRi technology (Zuberi et al., 2017b; Sharma et al., 2019).

### Biofilm Degradation by Electrochemical Method

**FIGURE 6:**
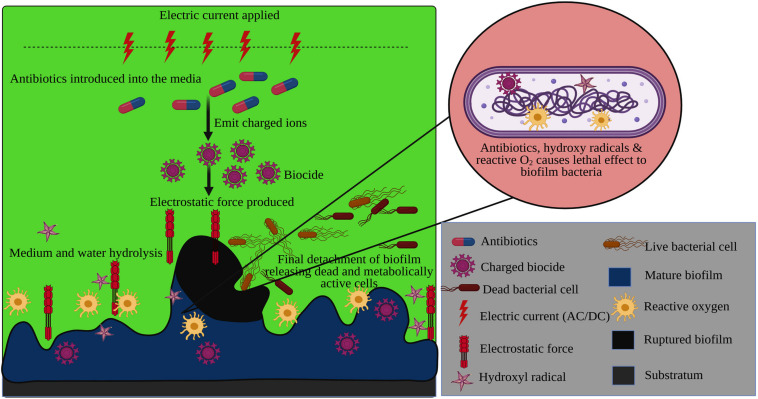
Electrochemical degradation of bacterial mature biofilm using the electrostatic force generated by an electric current with antibiotics.

The electrochemical method is one of the striking and promising strategies employed to put forth a great hindrance in bacterial biofilm formation. The electrochemical approach is the combinatorial effect of applying the lower dose of antibiotics in a weak electric field to disintegrate the biofilm formation or mature biofilm (Figure 6), which is also denoted as the ‘Bioelectric effect’. Several reports acknowledged that the electric potential lowers the antibiotics dosage to inactivate the biofilm and exerts a lethal effect on the biofilm organisms.

A contrary report has been stated by Shirtliff et al. (2005) when eliminating biofilm formation by mixed-species underwater. According to the application field, diverging hypothesis has been suggested for employing the electrochemical method to disperse the biofilm formation. The underlying principle behind electrochemical approach is that the antimicrobial binding and transport towards the biofilm matrix are enhanced due to electrostatic force under direct current and thereby it augments the efficacy of biofilm detachment (Blenkinsopp et al., 1992; Van Der Borden et al., 2004). Owing to the electric field, the media’s hydrolysis occurs, resulting in the release of charged ions and hyperoxygenation with thermal stimuli (Del Pozo et al., 2008).

Usually, it is very tedious for the antibiotics to penetrate the biofilm matrix. Under the influence of the electrical field, the antimicrobial agents lead to the discharge of the biocide ions attributed to the alteration in biofilm permeability. The influx of those biocide ions into the biofilm matrix consequentially inactivates the biofilm. It destroys the bacterial cells via electrophoresis and electro-osmosis even at a low concentration (Chang et al., 1995; Stewart et al., 1999). It has been clear evidence from the study of Blenkinsopp et al. (1992) that the electric current does not have any such effect over the biofilm unless it exhibits a synergistic action with the antimicrobial agents. The hydrolysis of water and the pH change in the electrical impulses expresses the high production of oxygen molecules contributing to the improved level of minimal inhibitory concentration, thereby increases antibiotic susceptibility among biofilm and drug resistant bacteria (Borriello et al., 2004; Del Pozo et al., 2009b; Wolfmeier et al., 2018). Furthermore, the hydrated ions create an electrostatic repulsive force that aids in detachment of the biofilm from the substratum surface (Poortinga et al., 2000).

The list of other significant factors related to the bioelectric effects also depends on the voltage and electric current during the electrical stimulation as it affects the cell membrane, cellular process, behavior, and electrophysiology (Sabelnikov et al., 1991; Hancock and Rozek, 2002). Certain research suggested implementing either Alternating Current (AC) or Direct Current (DC) (Stewart et al., 1999; Del Pozo et al., 2009a) or even both (Kim et al., 2015) to produce electrical impulse for bioelectric effect with the low dosage of antibiotics. In which, the AC contributes to the direct electrostatic force, and the DC attributes to the increased permeability as a result of charged molecules vibrations. In an *in vivo* experiment of Sultana et al. (2015), a drastic reduction of the biofilm has been observed. The biofilm of *Acinetobacter baumannii* has been developed on the porcine explant, which is superimposed with the electrochemical-scaffold. When an electric field is introduced using Ag/AgCl electrodes at the constant potential of about 600mV_Ag/AgCl_, the biofilm formation is much reduced due to the production of H_2_O_2,_ consequentially resulting in the synthesis of hydroxyl radicals leading to the lethal effect of the cell. In an another report using *P. aeruginosa* with a similar experimental setup employing glass-bottomed petri dishes as a biofilm model, a 10^5^-fold reduction in the biofilm formation has been demonstrated (Sultana et al., 2016). The action of plasma under low current influences a decline in the EPS intensity surrounding the bacterial cells resulting in low cellular adhesion (Kovalova et al., 2016). Another innovative technique for eradicating the biofilm employing the electrochemical method is electrospray. The dispersion of liquid from the high energy potential is carried out to obtain a sterile polymer surface devoid of biofilm (Kovalova et al., 2014).

### Surface Modulation of Bacterial Adhesion

There are numerous series of issues are found in marine, medical, food, and industrial fields due to biofilm formation. Especially in the marine environment, antifouling has attracted the utmost importance in recent years. Because biofouling created a severe threat in the marine environment and consequentially marine industries faced tremendous challenges. Biodeposition leads to the alteration of the nutritive source in the surrounding ecosystem, resulting in the disarray of the ecological niche in the benthic zone (Weise et al., 2009). Numerous studies have been carried out in search of innovative and novel technologies regarding the antifouling components. Antifouling compounds prevent or counteract the buildup of barnacles and other deposits, including microbial biofilms on the surface undersea. Dusane et al. (2011) demonstrated an antifouling compound from the marine strain *S. marcescens*, producing glycolipid surfactant that inhibits certain biofouling marine bacterial species such as *Bacillus pumilus*, *Candida albicans*, and *P. aeruginosa*.

Perhaps, the discovery of meticulous interaction outlay between the biofouling microbes and its substratum gives an alternative strategy against the biofouling by marine micro and macro-organisms. Modulation of the substratum or the surface to which the microbes attach is one interesting approach that has an impact over a few years in this field. Rogers et al. (1994) has sorted out various surface materials in the biofilm formation. The biocides recovered from certain microbes are implemented to coat on the surfaces to avoid the formation of biofilms, especially in the field of medicine. Due to the toxicity and other limitations, many regulations inclusive of European Union, Biocidal Products Regulation has been laid and restricted many biocides applications (Norcy et al., 2017). Tributyltin formulations are widely used in paints in earlier days, but their usage has been prohibited owing to their toxic nature towards the marine ecosystem. Instead, natural potent biofilm inhibitors have been focused. Multispecies biofilm formed by *E. coli*, *S. aureus*, and *P. aeruginosa* have been successfully eliminated using this technology by coating silver oxynitrate effectively (Lemire et al., 2017). Further, the numerous *in vitro* studies using silver coating materials have been extensively studied by various researchers (Lansdown, 2006; Stobie et al., 2008; Lemire et al., 2015; Kalan et al., 2017).

In the case of food industries, coating of the surface using non-toxic, nonstick components like silicones and fluoropolymer derivatives are preferred, because of its ability to form non-porous surface due to the association of hydrophobicity along with low surface free energy as well as the microroughness (Sadekuzzaman et al., 2015). Other attention grabbing criteria are the absorption of nanoparticles to the surface prevents the formation of biofilm. Such implementation has a vast impact in recent years in medicine and domestic purposes like pipelines, etc. The nanoparticles attach to the microbial surface, providing them with a larger surface area and thereby react with the protein and cellular DNA, eventually resulting in the inhibition of DNA replication and gene expression (Yamanaka et al., 2005; Sadekuzzaman et al., 2015). Hetrick et al. (2009) has demonstrated the biofilm inhibitory potential of nanoparticles embedded materials towards the pathogens such as *E. coli, P. aeruginosa*, *S. aureus*, and *S. epidermidis*. More, coating certain components like antibiotics, quaternary ammonium salts, polyethylene glycol, and silver ions towards the surface keeps away from bacterial colonization by weakening the cell membrane and the cellular activities (Park et al., 1998).

The application of bacteriocin is a promising approach for the control of biofilms due to their biofilm inhibitory property. Further, the biofilm formation of *Listeria monocytogenes* on the stainless steel has been destroyed using the bacteriocin derived from *Lactobacillus sakei* (Winkelstroter et al., 2011). Certain varieties of bacteriocin affect bacterial adhesion and biofilm formation even at the sub inhibitory concentration. The bovicin HC5 and nisin are the bacteriocin, which targeted the microbial cellular attachment by varying the microbial cell’s hydrophobicity and the substratum at the sub-inhibitory concentration (de Jesus Pimentel-Filho et al., 2014). This study also identified that bacteriocin inhibits the expression of *icaD*, *fnbA*, *clfB*, and *rnaIII* genes related to biofilm formation in *S. aureus*. The indwelling medical device surfaces coated with bactericidal compound like aryl rhodanines has prevented the biofilm development (Chung and Toh, 2014). Hence, the surface modulation allows the prevention of bacterial adhesion towards the substratum and thereby sets an obstacle for biofilm formation.

### Disruption of the Cell Membrane by Antimicrobial Peptides (AMP)

Recent research works continuously highlight that among the various biofilm inhibition strategies, the possible use of AMP, also known as host defense peptides, may denote a promising approach (Batoni et al., 2011; Jorge et al., 2012). The biofilm is disturbed through the different AMP by the transmembrane pore mechanism, which will lead to the final condition of cell death. The study of Pulido et al. (2016) reveals that the total permeabilization effect was visualized by Confocal Laser Scanning Microscopy (CLSM) and Sytox Green permeabilization assay, where the analysis of RN3 (5-17P22-36), an AMP, at higher concentration established the permeabilizing effect in biofilm cellular population. Indeed, the AMP action over the membrane depolarization and permeabilization facilitating the antimicrobial and biofilm inhibitory activities.

Interestingly, certain AMP binds to the bacterial cells of the biofilm structure, encouraging its cellular agglutination and membrane interaction. The peptides reorganization of the membrane determinants for the lipopolysaccharides lie at the N-terminus portion on coding sequence and thus expresses a higher specificity for the affinity towards the lipopolysaccharides on the cell membrane of Gram-negative bacteria (Bhunia et al., 2009; Torrent et al., 2009). The mechanism of AMP has been described in the models named ‘carpet’, ‘barrel-stave’ or ‘toroidal-pore’, which highlights the factors such as cationic charge, amphipathicity, amino acid composition, and size perusing the peptide attachment, translocation, and altering membrane permeability through an alternation in cytoplasmic membrane configuration.

In yet another report, the cationic peptide interaction towards the Gram-negative bacteria occurs to the anionic surface with the lipopolysaccharides layer on the outer membrane. It disturbs the structural configuration of the cell membrane, promoting the cellular leakage causing cell death (Moore et al., 1986). Further, Zhang et al. (2000) has accepted this view, insisting that the AMP interacts to the specific divalent cationic binding site at the lipopolysaccharides of the outer cell membrane bringing up the transposition through self-promoted uptake. Peptides are rendering interaction with the cell membrane based on its charge moiety and its hydrophobic interactions. Membrane targeting peptides like RT2, KT2, and magainin II enables their hydrophobic portion to interact with the anionic moiety in the lipid head of *E. coli* cell membrane and thereby launches them in the hydrophobic core (Anunthawan et al., 2015). Therefore, the structure, shape, and design of AMP are crucial for the electrostatic interaction to interrupt cell membrane and biofilm.

### Antimicrobial Lipids (AML) as Biofilm Inhibitors

Antimicrobial lipids (AML) are known as single-chain lipid amphiphiles, including fatty acids and monoglycerides (Verderosa et al., 2019). Later the 1,800s, after Koch and his colleagues first reported the growth inhibitory effects of soap, the antibacterial properties of fatty acids in soap had been identified and subsequently shown to inhibit *B. anthracis* growth, the causative agent of anthrax (Thormar, 2011). The antimicrobial potential of monoglycerides and fatty acids has been continuously exposed against various pathogens (Desbois and Smith, 2010; Desbois, 2012; Li et al., 2013; Karthikeyan et al., 2014; Wang et al., 2020). It is well understood that AML function through several pathways, such as enhanced membrane permeability, form temporary or permanent membrane pores, target the bacterial surface signal transduction system, electron transport chain destruction, cell lysis, and bacterial enzyme inhibition (Hyldgaard et al., 2012; Schlievert and Peterson, 2012; Verderosa et al., 2019).

**FIGURE 7:**
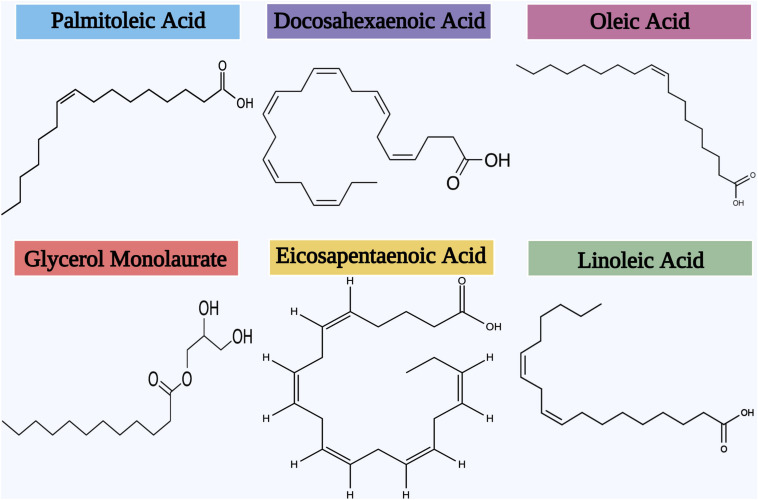
Chemical structures of the different AML that inhibit biofilm formation.

Several studies have continuously stated the biofilm inhibitory efficacy of AML at low doses against various bacterial biofilm formations (Figure 7). Oh and Marshall, 1995 has first explored the biofilm inhibitory efficacy of AML (Glycerol monolaurate) against *L. monocytogenes* biofilm formation. Glycerol monolaurate (GML) has antimicrobial or immunomodulatory effects, a fatty acid composed of glycerol and lauric acid (Witcher et al., 1996; Vetter and Schlievert, 2005). GML is currently used as a dietary and cosmetic ingredient, which is generally recognized as safe (GRAS) by the U.S. Food and Drug Administration. Schlievert and Peterson (2012) also validated the biofilm inhibitory efficacy of GML against *Haemophilus influenzae* and *S. aureus* biofilm formation. They have endorsed the bactericidal effects of GML on the mature biofilm formation of both bacterial pathogens. Recently, Lopes et al. (2019) substantiates the biofilm inhibitory potential of GML nanocapsules against *P. aeruginosa* biofilm by Atomic Force Microscopy.

A study made by Harvey et al. (2019) has revealed the biofilm inhibitory efficacy of antibacterial lipids on the cariogenic organism *Streptococcus mutans* by Fluorescent Microscopy using Alexa Fluor^®^ 647 and SYTO^®^ 9-labeled dextran conjugate. Further, the biofilm development of Gram-positive bacterial pathogens such as *S. epidermidis, S. aureus*, and *S. mutans* has been effectively inhibited by some unsaturated fatty acids such as oleic acid, linoleic acid, and palmitoleic acid (Yuyama et al., 2020). The inhibitory efficacy of the two fatty acids includes docosahexaenoic acid (DHA) and eicosapentaenoic acid (EPA) against *Fusobacterium nucleatum* and *Porphyromonas gingivalis* biofilms were recently studied by Sun et al. (2016). Obtained results of this study displayed that both DHA and EPA significantly eradicated the mature biofilm of *P. gingivalis* to the level of 61 and 47%, respectively. Further, both these two fatty acids were tested for action against *S. mutans* biofilm formation in a follow-up publication by the same research group (Sun et al., 2017). It was observed that both EPA and DHA significantly weakened the outer membrane of residing biofilm cells and thereby decreased the thickness of biofilm in *S. mutans*.

### Degradation of the EPS Matrix of Biofilm

The major components of the EPS matrix of biofilm are the polysaccharides, proteins, and nucleic acids (e-DNA), and thus one of the biofilm inhibition strategies includes attack over the biofilm matrix. The biofilm degradation is possible by disrupting the EPS matrix components. The biofilm EPS matrix components vary according to the microbial strain, age, and other environmental factors like pH, oxygen tension, and nutrient abundance (Flemming and Wingender, 2010). Lipopolysaccharide, alginate, Psl (Polysaccharide synthesis locus), and Pel (Pellicle) are the major EPS of the *P. aeruginosa* biofilm (Rabin et al., 2015). Other significant mechanisms regarding the action over the dissolution of the EPS matrix of biofilm include alginate lyase, DNase, and hydrolase-based approach. However, Dispersin B (DspB) protein acts upon the biofilm to disperse the EPS matrix in *Actinobacillus pleuropneumoniae* (Kaplan et al., 2004b). Similarly, DspB protein derived from the *A. actinomycetemcomitans* dissolute the biofilm matrix of *S. epidermidis* (Kaplan et al., 2004a,b). Further, DspB protein also affects the linkage of the glycosidic bond in the EPS matrix’s polysaccharide, and, hence the biofilm architectures are detached (Izano et al., 2007). Besides, certain phages and phage-derived enzymes like polysaccharide depolymerase can invade the EPS matrix and demolish the biofilm architecture (Sadekuzzaman et al., 2015).

The study of Powell et al. (2018) revealed the disruption of biofilm matrix through the implementation of alginate oligosaccharide (OligoG). The effect has been visualized using Lectin and ConA staining with CLSM imaging. The effects visualized by Stokniene et al. (2020) in CLSM images were similar to what was revealed in previous experiments: CLSM imaging visualized the OligoG and colistin conjugates disrupting the *P. aeruginosa* biofilm matrix. On the other hand, the interaction of divalent ions like Ca and Mg ions brings about the variation in EPS matrix through OligoG as these ions play a vital role in regulating the association of EPS and e-DNA in the mucoid matrix (Flemming and Wingender, 2010). These ions overcome the electrostatic repulsion of the negatively charged biofilm components and maintain biofilm physiology (Govan and Deretic, 1996; Whitchurch et al., 2002). It has been proved that Ca ions possess the efficacy in the stability and maintenance of the biofilm of marine species like *V. cholerae* (Kierek and Watnick, 2003). Furthermore, OligoG also influences the thickness of the biofilm (Strathmann et al., 2002).

### Inhibition of Alarmone Scheme

Under stress conditions like nutrient depletion, bacteria exhibit the stringent response, which is considered to be a crucial part for the synthesis of certain molecules in the alarmone scheme such as guanidine 3′diphosphate 5′triphosphate (pppGpp) and guanidine 3′5′bis-diphosphate (ppGpp), which are jointly expressed as (p)ppGpp through the cassette of RelA and SpoT. In Eubacteria, the stringent response is regulated by the signal molecule (p)ppGpp, which is produced by RelA and hydrolyzed by SpoT (Metzger et al., 1989), especially in Gram-negative bacteria and in Gram-positive bacteria with the aid of bifunctional enzyme, Rel/Spo for both the hydrolysis and synthesis (Wendrich and Marahiel, 1997). These molecules attribute the stringent response mediation that regulates the biofilm formation in *E. coli* and *S. mutans* (Lemos et al., 2004; Aberg et al., 2006).

The AMP 1,018 degrades alarmone ((p)ppGpp) signal by acting as the biofilm inhibitor. The biofilm inhibitory efficiency of the peptides 1,018 has been reported in earlier study (De La Fuente-Nunez et al., 2014). In the same study, the genes conferring the synthesis of alarmone (p)ppGpp signal in *P. aeruginosa* have been identified as *relA* and *spoT*. The gene expression and repression suggesting their part in the biofilm formation, maintenance, and outcome of the downregulation of those genes have also been documented through the *in vitro* studies of mutant deficient with the alarmone (p) ppGpp signal. The peptide 1,018 has more potential than the conventional antibiotics as it only suppresses the synthesis of alarmone (p) ppGpp signal rather than degradation. In addition, certain antibiotics have also been reported to affect the alarmone scheme, which eventually acts upon the alarmone (p) ppGpp signal synthesis, leading to adaptive resistance (Ikehara et al., 1985; Gilbert et al., 1990).

The exclusive investigation of He et al. (2012) provides insight into the regulation of the biofilm forming genes during stringent response in *V. cholerae*. It has been confirmed that due to the insufficiency in the synthesis of alarmone (p) ppGpp synthases, the shortage in the biofilm formation occurs. The stringent response is liable for certain regulatory factors responsible for the expression of *relA*, *spoT*, and *relV* in *V. cholerae*. It has been proved that the regulation of alarmone (p)ppGpp signal system of *vpsR* and *vpsT* in *V. cholerae* is accomplished through the transcriptional factor *rpoS*. Indeed, the sole source of alarmone (p) ppGpp synthase essential for the activation of the biofilm formation gene *vpsT* as it depends on RelA. The investigation of a novel compound named relacin has been designed to hold back the synthesis of alarmone (p)ppGpp signal molecules by inhibiting the RelA as it prompts the stringent response (Wexselblatt et al., 2012). During environmental stress like nutrient starvation, RelA induces the stringent response further affected by relacin and thereby down-regulated the biofilm formation in bacteria. Due to the reduction in alarmone (p) ppGpp synthesis, there is a low level of inosine monophosphate dehydrogenase synthesis, leading to low GTP consequences. The pyrophosphate’s ribosomal transfer describes the alarmone (p) ppGpp signal synthesis from ATP to GTP/GDP. Thus the relacin plays a major role in the biofilm formation pathway and other developmental pathways in *B. subtilis*.

Other peptides like LL-37, derived from natural peptides like human cathelicidin and 1,037, a synthetic cationic peptide, have also been reported for their biofilm inhibitory activity in earlier studies (Overhage et al., 2008; De La Fuente-Nunez et al., 2012). The other peptide includes the protease-resistant D-enantiomeric peptides DJK-5 and DJK-6 are noteworthy in describing the antagonistic activity against the biofilm formation in *P. aeruginosa* through inflowing into the biofilm cells and by degrading the intracellular nucleotides (p) ppGpp even at the low concentration range of 0.5–0.8 μg/mL below the MIC (De La Fuente-Nunez et al., 2015). Further, their variation and similarity in structure, function, and association are attributed to immune-modulatory, antimicrobial, antibiofilm, and anticancer properties. The AMP, which exerts a distinctive mode of action other than the conventional antimicrobial agents, are suggested for the biofilm inhibitory activity by hindering the alarmone system.

### Enzyme Mediated Biofilm Control

Certain enzymes are mediating the disruption of the biofilm of various bacterial species. Nevertheless, the bacteria themselves would synthesis certain endogenous matrix-degrading enzymes like glycosidases, proteases and DNase as it may induce the dispersion of the biofilm. DNase has been known for its biofilm inhibitory activity against Gram-positive and Gram-negative bacteria (Tetz et al., 2009). Further, DNase involve in breaking down of phosphodiester linkage in the backbone of e-DNA molecules in the formed biofilm, as the e-DNA is essential for the initial attachment and aggregation of the EPS onto the surface to make it an intact biofilm for a more extended period. Moreover, the e-DNA is one of the necessary factors for the biofilm formation, stability, and regulation (Das et al., 2010; Periasamy et al., 2012; Chagnot et al., 2013).

The synergistic action of DNase with metronidazole antibiotic established the disintegration of the biofilm formed by *Gardnerella vaginalis* at 100 μg/mL concentration. The report of Eckhart et al. (2007) also provided evidence for the suppression of biofilm through DNase in *P. aeruginosa* and *S. aureus*. For the first time, Monnappa et al. (2014) have been reported the extracellular protease and DNase from a host-independent *Bdellovibrio bacteriovorus* restrain the biofilm of Gram-positive bacteria especially, *S. aureus*. Furthermore, DNase (NucB) used as the feasible tool for dispersing e-DNA within the biofilm’s EPS matrix has been depicted by Nijland et al. (2010). Similarly, the study of Bugli et al. (2016) suggested the concentration of 100 mg/L DNase can able to eliminate the biofilm formed by *Helicobacter pylori*, while Torelli et al. (2017) gives insight on the DNase and alginate lyase as effective in disintegrating the biofilm of *Enterococcus faecalis* and *E. faecium* by acting over the EPS matrix of biofilm.

A significant biofilm inhibitory effect of proteases has been extensively described by Mukherji et al. (2015). The study of Hangler et al. (2009) discloses that Esperase HPF, a protease efficacy in preventing the biofilm of the species such as *Dokdonia donghaensis, Shewanella japonica*, *Microbacterium phyllosphaerae*, and *A. lwoffi*. Further, the commercially available proteases such as proteinase K, trypsin, and chymotrypsin (serine proteases category), and serratiopeptidase, carboxypeptidase A (metalloproteases category) have been reported for their biofilm inhibitory potential against *Staphylococcus* spp. (Artini et al., 2013). Craigen et al. (2011) has revealed the inactivation and removal of biofilm formation by *S. aureus* with the aid of alpha-amylase. Alginate lyase is a potent enzyme involved in the dissolution of biofilm of certain bacteria (Germoni et al., 2016). Many studies such as Rahman et al., 2010; Singh et al., 2011; Wang et al., 2013; Daboor et al., 2019 have indicated that the marine bacterium serves as a renowned source for the production of enzyme alginate lyase, as a powerful agent in disassembling the biofilm exerted by pathogens. It may be related to the fact that marine bacteria are exposed to substantial quantities of the alginate present in their surroundings (Zhu et al., 2016). The enzyme alginate lyase is recovered from the marine bacterium *Pseudoalteromonas* active in exhibiting biofilm inhibitory activity against *P. aeruginosa*, *E. coli*, and *S. enterica* (Dheilly et al., 2010; Dufourcq et al., 2014).

### Mechanical Eradication of Biofilm Formation Through a Photodynamic Approach

The photodynamic approach is the recent innovative method adapted for the disintegration of biofilms. The underlying principle behind this approach is the implementation of photosensitizing molecules that absorb the light intensity of a specific wavelength and, by binding to the target cellular components like lipid, protein, and nucleic acid. It produces reactive oxygen radicals, which in turn will give rise to hydrogen peroxide, hydroxyl radicals and superoxide anion resulting in the lethal/toxic effect to the target. There are the latest attempts for evidencing biofilm inhibition in this regard (Darmani et al., 2018; Gholibegloo et al., 2018). The photosensitizer and the light source implemented are crucial for this kind of mechanical eradication of biofilms through photodynamic therapy. The frequently employed photosensitizer includes methylene blue, toluidine blue, or toluidine blue O. Other photosensitizer comprises 23H-porphine, tetrakis (1-methyl-pyridino)-21H, tetrakis (phenylthio)-29H, 31H-phthalocyanine, and tetra-p-tosylate salt (Collins et al., 2010; Junqueira et al., 2012; Sadekuzzaman et al., 2015).

The absorption spectrum of methylene blue is at 664 nm, while toluidine blue is at 638 nm and lies within the UV-visible range of 600–1000 nm wavelength. Though methylene blue photosensitizer is effective against various bacterial pathogens, it has more potential to eliminate Gram-positive bacteria. It is due to the reason that it constitutes additional effectiveness in transferring the negative charge towards the target cell with a negative charge cell wall owing to the presence of teichoic acid (Mirouze et al., 2018; de Oliveira et al., 2019). Although light emission is accomplished through the diode laser while employing toluidine blue as photosensitizer (Zanin et al., 2006), the study of Soukos et al. (2000) has reported that the light emission from helium/neon laser light employing toluidine blue as a photosensitizer in eradicating the 95% of oral biofilm formation. Furthermore, Andrade et al. (2013) has suggested that pre-radiating time over the target influences the elimination of the microorganism. Considering future viewpoints, more attempts have to be made to establish the complete knowledge and detailed mechanism of action for successful photodynamic therapy in eradicating bacterial biofilm formation.

### Interruption/Down-Regulation of Molecular System of Biofilm Formation

Transcriptional regulation of a gene is far most important for gene expression to occur. The regulatory proteins and regulatory binding sites are also promising factors for regulatory machinery. The detailed study regarding gene expression profiling will pay for the appropriate up-regulation or down-regulation mechanism. Considering such approach, the biofilm formation can be interrupted or blocked through the downregulation of biofilm synthesis genes via certain components. Nal-P-113 is an AMP that acted against the biofilm formation of *P. gingivalis* W83 by deregulating the genes that confer biofilm formation, thereby controls the infection (Wang et al., 2017). These authors evaluated and described that when the gene *PG0282* and *PG1663* gets down-regulated through the mediation of Nal-P-113 peptide, it directly influences the ABC transporter, which plays a critical role in the biofilm formation machinery.

Upregulation of the ABC transporter is necessary for the initial biofilm formation when evaluating the *potB* gene coded for ABC transporter in *P. putida* (Sauer and Camper, 2001). This view has also been accepted by Hinsa et al. (2003). Later they have evaluated the association between the biofilm formation and the ABC transporter system. Though *lapEBC* cassette or lap gene encoded for the ABC system, the mechanism and the genes coding vary between species and strain. Furthermore, down-regulation of certain genes reduces the biofilm formation, as narrated in a few studies (Whiteley et al., 2001; Overhage et al., 2007). The study of De La Fuente-Nunez et al., 2012 highlighted the genes conferring swimming and swarming motility to be down-regulated to influence biofilm elimination using the AMP 1,037.

Several earlier studies also illustrated that the down-regulation of certain genes including *flgB*, *nirS*, *norC*, and *nosZ* (which are responsible for anaerobic biofilm formation), *fimX* (encoding for Type IV pilus assembly), *rhlB* (regulating QS and involved in the rhamnolipid synthesis), *lecB* (involved in the fucose binding of lectin) hinders the biofilm formation in different bacterial pathogens (Schuster et al., 2003; Johansson et al., 2008; De La Fuente-Nunez et al., 2012). The novel gene *rsaL* has been earlier demonstrated by De Kievit et al. (1999), which involved in the synthesis of the harmful regulatory protein that impacts the suppression of *lasI* gene expression. The gene product of *rsaL* has a negative transcription regulation of *lasI* operon in *P. aeruginosa*, thereby obstructing the QS cascade, leading to biofilm inhibition. The other approaches include altering the QS signaling cascade to prevent the biofilm formation is CRISPRi technology for deregulating the *luxS* gene expression (Seed et al., 1995; Lonn-Stensrud et al., 2009; Zuberi et al., 2017b), which is involved in QS signaling and fimbriae associated gene (*fimH*) expression, so that, it has an influencing effect to suppress the biofilm formation (Zuberi et al., 2017a).

## Conclusion and Future Prospective

In nature, several bacteria live in the form of biofilms. For the medical community, biofilms constitute a serious problem since not only they are linked with numerous infections in humans, but they are often particularly challenging to handle, because of their tolerance to antibiotics and immune responses. Biofilm mediated infections are difficult to control due to their complexity and increasing antibiotic resistance. It is necessary to prevent their surface colonization to restrict biofilm development, as this is the first step in the formation of biofilms. In this review, we have discussed several emerging strategies and potential perceptions for developing enhanced therapeutics to control biofilm mediated bacterial infections. The alternative approaches for preventing biofilm formation in the medical devices or marine environment are also explored in depth, with special emphasis on surface modulation of bacterial adhesion. Overall, all these recent therapeutic biofilm inhibition strategies can open up new prospects for controlling biofilm development in diverse sectors. Future prospective of improved biofilm eradication strategies may aim to commercial intake of certain biofilm inhibitors like enzymes, AMP, AML, and QS inhibitors would make it possible as a tremendous tool to achieve the target. Nevertheless, in-depth research is necessary to clarify the effect of these biofilm inhibitors during biofilm infection in the host and prove their applicability to humans. Meanwhile, biofilm inhibitors may not cause antibiotic resistance; they have a lot of promise in the future for treating biofilm based infections in healthcare settings.

## Author Contributions

RS, SS, and LX conceived the concept of the review. RS, SS, PP, and MG drafted the manuscript. RS prepared the figures. RS and LX coordinated the work and acquired funding. RS, SS, MD, and LX reviewed and edited the manuscript. All authors contributed to the article and approved the submitted version.

## Conflict of Interest

The authors declare that the research was conducted in the absence of any commercial or financial relationships that could be construed as a potential conflict of interest.
